# Assessing the use of contraceptives by female undergraduate students in a selected higher educational institution in Gauteng

**DOI:** 10.4102/curationis.v38i2.1535

**Published:** 2015-11-26

**Authors:** Maria H. Coetzee, Roinah N. Ngunyulu

**Affiliations:** 1 Department of Nursing Science, University of Pretoria, South Africa

## Abstract

**Background:**

Unplanned pregnancies amongst students at higher education institutions are a major concern worldwide, including South Africa. Apart from various social and psychological challenges, unplanned pregnancies affect students’ objectives of achieving academic success. Research undertaken in the United States of America (USA) indicates that around 80% of female students in institutions of higher education between ages 18 and 24 are sexually active.

**Objectives:**

To assess and describe the use of contraceptives by undergraduate female students in a selected higher educational institution in Gauteng.

**Method:**

A cross-sectional, descriptive, quantitative design was used. A total of 400 female undergraduate students were requested to respond to a self-administered questionnaire. Stratified random sampling was used to select the participants. They were selected systematically from two campuses. Data were entered using an excel sheet at the Department of Statistics, and analysed using the Statistical Analysis Software programme, (SAS version 9.3), of the Department of Statistics’ higher educational institutions.

**Results:**

A total of 74% females indicated they were sexually active, 79% of whom reported using contraceptives. The most common used methods were oral contraceptives at 38%, and 25% for male condoms. The most commonly known methods were condoms at 84%, and the oral contraceptive at 68%. The knowledge of condom use to prevent sexually transmitted diseases was high at 91%.

**Conclusion:**

Inadequate knowledge and awareness on some contraceptive methods was found. Thus, educational programmes to increase students’ knowledge on the use of all contraceptive methods are urgently needed.

## Introduction

### Background

Throughout the world, female students are exposed to the risk of unplanned pregnancies as a result of ineffective or non-use of contraceptives (Dreyer 2012:6; Maja & Ehlers 2004:43). This may result in failure to complete their education, inability to maintain gainful employment and making independent marital decisions (Maja & Ehlers 2004:43). Young students’ sexual activities are a communal, municipal and public health concern. These activities, especially pre-marital sexual activities, seem to be increasing amongst higher educational institution students in countries such as Asia and Africa, because of factors such as rapid urbanisation and exposure to mass media (Mehra *et al.* 2012:1).

Studies in Nepal and in South Africa (Adhikari 2009:2; MacPhail *et al.* 2007:5) reported that the lack of adequate knowledge and awareness of effective contraceptive use amongst higher educational female students, results in the non-utilisation of contraceptives. This eventually contributes to high unplanned pregnancy rates. It is estimated that it contributes to about 8 to 30 million annual pregnancies worldwide (Adhikari 2009:2; MacPhail *et al.* 2007:5). Approximately 210 million pregnancies occur annually across the world, of which 75 million (or about 36%) are unplanned or unwanted (Adhikari 2009:2). This is supported by international studies that reported that students between 18 and 24 years have one of the highest rates of unplanned pregnancies. The lack of effective knowledge concerning contraceptive use results in an increase in unplanned pregnancies (Bryant 2009:12; Trieu *et al.* 2011:431).

In research studies conducted worldwide amongst university students, several factors were identified as contributing to the non-utilisation of contraceptives. These were, amongst others, lack of knowledge and awareness, age, culture, ethnicity, religion, poor access to contraceptive services, peer pressure, sources of information, alcohol and substance abuse and lack of partner support (Ahmed *et al.* 2012:2; Golbasi, Tugut & Erenel 2012:81). Another study conducted amongst university students in the United States of America (USA) estimated that regular contraceptive use can prevent about 12 million unwanted pregnancies every year (Ersek *et al.* 2011:497).

In a study amongst 15 to 24 year old South African women, it was estimated that only 52.2% of sexually experienced women are using contraceptives (MacPhail *et al.* 2007:3). Because of the fact that 80% of undergraduate students at higher educational institutions are sexually active, it is vital that they have access to safe, accessible and adequate contraceptive services (Bryant 2009:12). Dreyer (2012:6) suggests that the main reasons for women not utilising or discontinuing the use of contraceptives are side effects, lack of knowledge about different methods available, or lack of interest in utilising it.

In the study amongst students in Durban, South Africa, Roberts, Moodley and Esterhuizen (2004:441) suggested that an increase in the use of emergency contraceptives might reduce the number of unplanned pregnancies. However, as a result of the lack of knowledge and awareness thereof, the family planning services were underutilised (Roberts *et al.* 2004:441).

The study intended to assess and define contraception use amongst students in a higher educational institution. The results can be used to improve the students’ knowledge that would enable them to make informed decisions regarding contraception use and prevention of unplanned pregnancies.

## Problem statement

Every year throughout the world, the rate of unplanned pregnancies amongst students at higher educational institutions continues to increase (Table 1). This is despite the availability of free contraceptives and emergency contraceptives offered by student health centres at higher educational institutions (Brunner Huber & Ersek 2009:1069; Maja & Ehlers 2004:43).

**TABLE 1:** The unplanned pregnancy rate from 2009 to 2013 at the selected higher educational institution.

Campus	Year			
	2009	2010	2011	2012	2013
**Campus A**					
A – number of pregnant students	59	28	42	39	86
A – number of female students	11849	12707	12709	12367	12822
A – percentage of pregnant students	0.50%	0.22%	0.33%	0.32%	0.67%
**Campus B**					
B – number of pregnant students	12	16	9	9	9
B – number of female students	1978	2208	2388	2504	2633
B – percentage of pregnant students	0.61%	0.72%	0.38%	0.36%	0.34%
**Both campuses**					
Number of pregnant students	71	44	51	48	95
Number of female students	13827	14915	15097	14871	15455
Percentage of pregnant students	0.51%	0.30%	0.34%	0.32%	0.61%

*Source*: The selected higher educational institution

Lack of awareness and knowledge on the use of contraceptives is associated with the failure of their utilisation (MacPhail *et al.* 2007:5). The high rate of unplanned pregnancies caused multiple challenges for academic institutions across the world. These challenges relate to high dropout rates by students, serious financial losses for academic institutions and an increased drain on public sector funds (Vermaas 2010:1).

### Aim of the study

The aim of this study was to assess and describe the use of contraceptives by undergraduate female students and to address the identified problem of unplanned pregnancies in a selected higher educational institution.

### Trends

In a study conducted on contraceptive use and attitudes amongst female college students, it was suggested that college students aged between 20 and 24 have one of the highest rates of unplanned pregnancies because of the lack of contraceptive use (Bryant 2009:12). Several studies conducted internationally and in South Africa have found that a lack of knowledge of contraceptives may lead to an increased rate in unplanned pregnancies (Akintade, Pengpid & Peltzer 2011:72; Roberts *et al.* 2004:441).

## Research objective

The primary objective of the study was to assess the use of contraceptives amongst undergraduate female students.

## Definition of key concepts

**Assessment:** Determining the use of contraceptives (Oxford University 2004:26).

**Contraception:** A birth control method used by female undergraduate students between the ages of 18 and 24 to prevent a pregnancy (Bafana 2010:13).

**Higher educational institution:** The selected institution offering undergraduate programmes (*Higher Educational Act* No. 101 of 1997).

**Student:** All female undergraduate students between the ages of 18 and 24 who are registered at the selected institution of higher education (*Higher Educational Act* No. 101 of 1997).

**Unplanned pregnancies:** A pregnancy that was not planned by a female undergraduate student between 18 and 24 years of age, during the course of her studies (Ahmed *et al.* 2012:3).

## Contribution to field

The outcome of this study could be used by professionals and students to reduce the rate of unplanned pregnancies, by assisting students in making informed decisions regarding their sexual health. This may include the provision of a platform for effective intervention, and creating opportunities for higher educational institutions to improve their current sexual and reproductive health services.

Students can advance their academic career within a shorter period which would prevent a financial drain on higher education and public services. Less unwanted pregnancies may lead to substantial cost saving in terms of social spending by the South African government.

## Literature review

The high rate of unplanned pregnancies amongst students at higher education institutions has become a major concern worldwide (Zhou *et al.* 2012:1153). Several studies conducted internationally and in South Africa have found that a lack of knowledge on contraceptives may lead to an increased rate in unplanned pregnancies (Akintade *et al.* 2011:72; Roberts *et al.* 2004:441)

## Prevalence of unplanned pregnancies

Estimates of the global incidence of unplanned pregnancy and pregnancy outcomes were developed for the first time in 1995 and published more than ten years ago (Singh, Sedgh & Hussain 2010:241). At that time, about 38% of all pregnancies were estimated to be unplanned and more than half of these (22%) ended in abortion. Of the 208 million pregnancies that occurred worldwide in 2008, it is estimated that 41% or 86 million were unintended or unplanned, and 185 million of these took place in the developing world (Singh *et al.* 2010:241). In a study in 2009 amongst college students in Nepal, it was indicated that approximately 210 million pregnancies occur across the world 75 million or 36% of which are unplanned or unwanted. The higher rates of unplanned or unwanted pregnancies occur amongst university age women, with 60% being unplanned. This is supported by other studies that reported that almost half of the pregnancies in the USA are unplanned, mostly occurring amongst 18 to 24 year old women (Brunner Huber & Ersek 2009:1063; Eisenberg *et al.* 2012:479.e1). In more recent studies, the World Health Organisation (WHO) estimated that about 45% of pregnancies across the world are unplanned, unintended or unwanted, and that about half of them end in termination of pregnancy (Dreyer 2012:6). There is, therefore, strong evidence that at least half of all pregnancies worldwide are unplanned, and many occur amongst university age students between 18 and 24 years.

Every year in sub-Saharan Africa, about 14 million unplanned pregnancies occur, 44% of which are amongst women aged 15 to 24 (Hubacher, Ifigeneia & McGinn 2008:73). In a study by Mehra *et al.* (2012:110), it was suggested that 39% of the approximately 41% unplanned pregnancies worldwide occur in Africa. In another study conducted in South Africa, it was revealed that during the 2003 Demographic Health Survey, the occurrence of unplanned pregnancies was 61% (Bafana 2010:11).

## Contraceptive use

Contraceptive use prevalence in the world was estimated at 63% in 2000, with higher levels of use in developed countries at 70%, and in less developed countries at 61% (Bafana 2010:7).

In countries where the incidence of unplanned pregnancies was decreasing, there was an increase in the use of contraceptives (Singh *et al.* 2010:246). The use of both modern and traditional methods increased steadily throughout the world, indicating that women have not moved towards more effective methods (Singh *et al.* 2010:246). In a study conducted on contraceptive use and attitudes amongst female college students in the USA, it was suggested that between ages 20 and 24 they have one of the highest rates of unplanned pregnancies. It was indicated that 53.3% did not use contraception (Bryant 2009:14). Two reasons given for the pregnancies were contraceptive failure or incorrect use of contraceptives (Bryant 2009:16).

In 2000 it was estimated that Africa had the lowest rate of contraceptive use in the world at only 28% (Bafana 2010:7). In sub-Saharan Africa, approximately 14 million unplanned pregnancies occur and a fairly large proportion of unplanned pregnancies are because of poor use of short-term hormonal methods (Hubacher *et al.* 2008:73).

In research conducted amongst female students at the National University of Lesotho, it was revealed that at the age of 24 over two thirds of young South African girls are sexually active, wherein 50% had fallen pregnant and only half have ever used contraceptives (Akintade 2010:8).

Studies found that the overall prevalence of contraceptive use in South Africa, during the 2003 Demographic Health Survey, was 65%. (Bafana 2010:7). In a study conducted amongst higher educational students in Durban, it was suggested that an increase in the use of emergency contraceptives would reduce the number of unplanned and unwanted pregnancies, as well as the number of induced abortion (Roberts *et al.* 2004:441).

Some studies found that racial differences also occur in contraceptive use (Bafana 2010:7). Religion was found to influence the use of contraceptives (Akintade *et al.* 2011:76). In other studies it was suggested that family, friends and peers also have a big influence on the use of contraceptives (Mehra *et al.* 2012:8). It is estimated that regular contraceptive use can prevent 12.0 million unplanned and unwanted pregnancies every year (Ersek *et al.* 2011:497)

## Research method and design

### Design

A cross-sectional, descriptive, quantitative survey was used to assess the use of contraceptives. The population for female undergraduate students at campus A was 12 447, and 2545 at campus B. The population size for campus A was 111 participants and 106 participants for campus B. Stratified random sampling was used to divide the population in two strata, namely campus A and campus B. To obtain a representative sample, systematic sampling was applied to select participants. Basic ethical principles were applied during the entire study, such as beneficence, respect for human dignity and justice (Polit & Beck 2012:152–156).

### Materials

A structured questionnaire was used and included two sections. Section A included six items on social and demographic characteristics, and section B included 10 items on the use of contraceptives. Close-ended questions were used.

### Data collection method

A questionnaire was used to collect the data. Data were collected through structured questionnaires during June and July 2014. The questionnaires were emailed to the selected participants and, after two weeks, a follow-up email was sent to those participants who had not yet responded. A final email was sent out after four weeks to participants who had not responded, reminding them about the research. Respondents who had difficulty in completing the questionnaire online were kindly requested to visit their Student Health Services to complete the printed questionnaire (Abera & Tebeje 2009:38; Adhikari 2009:2; Akintade *et al.* 2011:73).

### Data analysis

Out of the 400 questionnaires distributed, 217 were returned, giving a response rate of 54.24%. Data were entered and analysed using the Statistical Analysis Software programme (SAS version 9.3), of the Department of Statistics for institutions of higher education. Descriptive statistical analyses were applied to present the results.

Frequency tables were generated for all variables, which were used for descriptive statistics based on frequencies and percentages. Graphs and pie-charts were generated when analysing results.

### Context of the study

The research was conducted amongst female undergraduate students between the ages of 18 and 24 at two selected campuses in a selected higher educational institution in Gauteng, South Africa. At campus A, approximately 1250 students are treated per month for primary health. Primary health care services are rendered five days per week by three primary health care sisters, one registered nurse and three medical doctors. At campus B, approximately 150 students are treated per month. Services are rendered by a primary health care sister three days per week and a medical doctor once a week for two hours respectively. The services provided are primary and preventative health care, reproductive health services, HIV voluntary counseling and testing services, dietary clinic, health education, handling of student emergencies on campus and the doctor’s clinic.

## Results

### Social and demographic characteristics of participants

A total of 217 female undergraduate students participated in the study. At campus A there were 111 and 106 at campus B providing a response rate of 54.24% for both campuses.

As indicated in Table 2, the participants’ age distribution was between 18 and 20 years and 21 to 24 years, making the mean and standard deviation for campus A, 20.71 and 1.47; and 20.71 and 1.45 for campus B respectively. A majority of participants at campus A, 92.79%, and 95.24% from campus B were single, whereas at campus A, 7.21% were cohabiting compared to 4.76% at campus B. At campus A 73.93% and 77.36% at campus B were black students. At campus A, 1.8% were Asian, 2.70% were mixed race and 21.56% were white people, whereas at campus B, 3.77% were mixed race, 18.87% were white people and 0.00% was Asian.

**TABLE 2:** The means procedure of the age of participants.

Campus	Mean age	Median age	Standard deviation	Minimum age	Maximum age
Campus A	20.71	21.00	1.47	18	24
Campus B	20.58	20.00	1.45	18	24

At campus A, 43.27% resided in a flat, 19.83% resided in their parents’ home, 26.07% stayed in a university residence and 10.82% in a university commune. At campus B, 58.49% stayed in the university residence, 20.75% resided in a flat, 17.92% in their parent’s home, and 2.83% in a university commune. The predominant religion of participants at both campuses was Christian, campus A at 92.72% and 96.23% at campus B. The rest of the participants, 7.28% at campus A and 3.77% at campus B, indicated various religions which included Hindu, Judism, Moslem and Scientology (Table 3).

**TABLE 3:** Social and demographic characteristics of respondents (*n* = 217).

Variable	Campus	Frequency	Percentage
**Age**			
18–20	A	53	47.71
21–24		58	52.29
18–20	B	58	54.72
21–24		48	45.28
**Marital status**			
Single	A	103	92.79
Staying together		8	7.21
Single	B	100	95.24
Staying together		6	4.76
**Ethnic group**			
Asian	A	2	1.80
Black people		82	73.93
Mixed race		3	2.71
White people		24	21.56
Asian	B	0	0.00
Black people		82	77.36
White people		4	3.77
White people		20	18.87
**Type of residence**			
Living in a flat	A	48	43.27
Parents home		22	19.83
**Variable**			
University residence	A	29	26.07
University commune		12	10.82
**Type of residence**			
Living in a flat	B	22	20.75
Parents home		19	17.92
University residence		62	58.49
University commune		3	2.83
**Religion**			
Christian	A	102	92.72
None of the above		8	7.28
**Religion**			
Christian	B	102	96.23
None of the above		4	3.77

### The use of contraceptives

The prevalence of contraceptive use amongst the respondents was high, and the majority indicated that they used some method of contraception. As presented in Table 4 the majority at both campuses was sexually active. Amongst the 70.96% of sexually active participants at campus A, 88.46% indicated they used contraceptives. At campus B, 76.92% indicated they were sexually active, with 68.75% using some method of contraception.

**TABLE 4:** Percentage of sexual activity and contraceptive use.

Campus	Sexually active (%)	Percentage of contraceptive use	Percentage of no contraceptive use	Total
A	70.96	88.46	11.54	100.00
B	76.92	68.75	31.25	100.00

*Source:* University Department of Statistics

As indicated in Table 5 the oral contraceptives were used by the majority of participants at campus A at 41.46% and 34.21% at campus B. The Depot Medroxy Progesterone Acetate (DMPA) injection was used by approximately 9.5% of respondents at both campuses, whereas 13.41% at campus A and 26.32% at campus B used the Norethisterone Enanthate (NET-EN) injection. A total of 5% at both campuses used abstinence as a contraceptive method and 25% at both campuses used condoms as a contraceptive method.

**TABLE 5:** Percentage of method of contraceptive used.

Variable	Campus A	Campus B
Oral pills	41.46	34.21
DMPA injection	9.76	9.21
NET-EN injection	13.41	26.32
Abstinence	4.88	5.26
Condoms	24.39	25.00
Emergency contraceptive	4.88	0.00
Hormonal patch	1.22	0.00

Results indicated that 61% at both campuses were using contraceptives consistently (every day) and 79% at both campuses indicated they used contraceptives correctly and as prescribed. At campus A, 5.26% and 29.41% at campus B indicated reasons for failure of contraceptive were fear of possible side effects. At campus A, 36.84% and 23.53% at campus B indicated failure as a result of not using it consistently and every day. Twenty-one point zero five per cent (21.05%) at campus A and 17.65% at campus B responded that failure was because of incorrect use. Twenty-one point zero six per cent (21.06%) at campus A and 5.88% at campus B indicated that failure was because of pregnancy, and 15.79% at campus A and 23.53% at campus B indicated that they were not sure why it failed.

## Ethical consideration

Prior to the research, approval from the Director of Student Affairs, and the Student Representatives Council were obtained. In addition, ethical clearance and approval for the study was obtained from the Research, Ethical Committee.

An official letter to request permission from the higher educational institution’s administrative office was obtained and permission was granted to conduct the research. A participant information leaflet and informed consent form for anonymous questionnaires were emailed to the selected participants before collecting data.

### Quality control

Validity and reliability were used to ensure quality control. Quality control was conducted during every stage of the study. Content and face validity was confirmed by the literature study, as well as by pilot testing the instrument prior to the study on women who were excluded from the actual study. Content validity of the research instrument was ensured by using standardised reproductive health tools as a guide during the preparation of the questionnaire and through consultation with reproductive health professionals. The instrument’s reliability was ensured as a result of the fact that the characteristics of the group for which it was developed were known.

## Discussion

### Social and demographic characteristics

The age range of participants was from 18 to 24 years. About 78.6% of respondents between 18 and 24 years were using contraceptives and almost 94% were single and formed a majority. Black students were the biggest ethnic group at 75.6% and the predominant religion was Christianity at 94%. Seventy four percent (74%) of the participants indicated they were sexually active, 79% of whom indicated they used contraceptives. This is in line with another study amongst female college students that revealed that 80% of higher educational females are sexually active (Bryant 2009:12).

### The use of contraceptives

As noted from the results, the method of contraceptive most commonly used was oral contraceptives at 38%, followed by the condom at 25%. Almost 40% of participants indicated that they did not consistently use contraceptives. A large proportion of participants, namely 79%, indicated they used contraceptives correctly. A smaller sample of 17% responded that a contraceptive method has failed them because of the fear of possible side effects.

### Implications

A major responsibility of health care providers at campus clinics is to educate students on sexual and reproductive health issues. Based upon results in this study and previous studies, educating females about preventing unplanned pregnancies can decrease some psychosocial challenges associated with having an unplanned pregnancy (Bryant 2009). Health care providers should assist female students to make informed decisions about contraceptive use by educating them on their concerns about side effects and the reliability of contraceptives to increase their use. Misconceptions about contraceptive methods, that is, that they cause cancer and weight gain can be decreased by accurate, appropriate health education on the methods, and assisting them in choosing a most appropriate method for their individual needs. This can increase the consistent use of contraceptives, and decrease the rate of unplanned pregnancies. By reducing unplanned pregnancies the population growth could slow down, thereby reducing government’s need to expand infrastructure at high costs. Nurses can improve quality care at community nursing clinics by providing culturally appropriate care which may increase client satisfaction with the clinic (Bryant 2009:16).

### Limitations

This study was conducted using respondents from two campuses at one higher educational institution in Pretoria, and is thus not necessarily applicable to other campuses or other higher educational institutions. As the data collection instrument addressed a number of questions that students may have felt were personal or sensitive in nature, it may be possible that they did not accurately respond regarding the use of contraceptives. Poor recall of information, as respondents may not have remembered all the details of their contraceptive use, may have led to bias. Some questions which were not identified during the pilot study may have been repetitive in nature and could have influenced the accuracy of the results. Another limitation was that the study was limited to female students. The study’s response rate was 55% which was of concern to the researcher.

### Recommendations

Programmes to increase young females’ knowledge of all contraceptive methods and their effective use are required. The improvement and consistent use of contraceptives was also recommended by another study (Frost, Lindberg & Finer 2012:107).

Health care workers should give adequate, accurate and detailed information about the possible side effects of contraceptives to decrease their inconsistent use. They could also request higher education students using the student health services about concerns or challenges in obtaining their contraception and work with them to solve them. They should also stress the importance of consistent contraceptive use, especially condoms, to prevent not only unplanned pregnancies but also sexually transmitted diseases, considering the low percentage of students using condoms found in this study. Considering that only 50.3% of females were familiar with emergency contraceptives, health care workers could educate females on how to obtain and use them in preventing unplanned pregnancies.

## Conclusion

The prevalence of contraceptive use by sexually active students in this study was high at 79%. However, inconsistent use of contraceptives is a major challenge. Females were aware of the benefits of contraceptives in preventing unplanned pregnancies; however they used contraceptives inconsistently as a result of being afraid of possible side effects. Overall, there was a limited awareness and use of emergency contraceptives. Consistent use of regular contraceptives and condoms should be emphasised to reduce not only unplanned pregnancies but also sexually transmitted diseases (Brunner Huber & Ersek 2009:1069).
